# Regulation of B cell tolerance by 129-derived chromosome 1 loci in C57BL/6 mice

**DOI:** 10.1002/art.23553

**Published:** 2008-07

**Authors:** Liliane Fossati-Jimack, Josefina Cortes-Hernandez, Peter J Norsworthy, H Terence Cook, Mark J Walport, Marina Botto

**Affiliations:** Imperial CollegeLondon, UK

## Abstract

**Objective:**

Systemic lupus erythematosus is a multifactorial disease with a strong genetic component. Previous studies have shown that a 129-derived chromosome 1 interval (*Sle16*) on the C57BL/6 (B6) background is sufficient to induce humoral autoimmunity. The aim of the present study was to elucidate the mechanisms by which this locus contributes to the loss of peripheral tolerance.

**Methods:**

Anti–single-stranded DNA (anti-ssDNA)–knockin transgenic mice (V_H_3H9R/Vκ8R and V_H_3H9R) were crossed with a B6 congenic line named B6.129chr1b that carries the *Sle16* locus. A parallel study of a gene-targeted animal, whose mutated gene is located within the *129chr1b* interval on chromosome 1, was also performed.

**Results:**

The combination of V_H_3H9R/Vκ8R with the *129chr1b* interval resulted in impaired B cell anergy, and transgenic IgM and IgG anti-ssDNA antibodies were found in the circulation. The presence of IgG2a^a^ anti-ssDNA and IgM^a^ anti-Sm antibodies in sera indicated that the autoreactive transgenic B cells underwent class switching and epitope spreading. The *129chr1b* locus appeared to have a dominant effect, since transgenic antibodies were also detected in mice carrying a single allele. The gene-targeted animals showed a similar phenotype.

**Conclusion:**

The presence of a single *129chr1b* locus on the B6 background impaired B cell anergy, prevented deletion of anti-DNA transgenic B cells, and induced receptor revision. The findings of this study also emphasize that the autoimmune phenotype observed in mice with targeted genes located on chromosome 1 may simply arise from epistatic interactions between the 129 and B6 parental strains.

Systemic lupus erythematosus (SLE) is an autoimmune disease that displays highly variable clinical features. The serologic hallmark of SLE is the production of autoantibodies directed against a wide spectrum of self antigens, especially nuclear components, such as DNA. Several lines of evidence indicate that both in humans and in animal models, SLE susceptibility is inherited as a multifactorial genetic trait (1). To identify the genetic components of SLE, linkage analyses have been performed in spontaneous lupus–prone mice, and these studies have led to the identification of several genomic intervals (2). Interestingly, a great proportion of the intervals detected are strain-specific, confirming the genetic complexity of the disease and indicating the presence of extensive heterogeneity in the genes that contribute to the pathogenesis of SLE.

However, 1 interval, located on the distal region of chromosome 1 in the mouse and on its orthologous region in humans, has shown strong linkage in several human and murine studies (3,4). One of the best characterized loci in this region is *Sle1b*, which was identified via linkage analysis of the lupus-prone NZM2410 mouse model (5). This locus, when expressed on the C57BL/6 genetic background (B6.*Sle1b*), can induce a relatively benign autoimmune disease characterized by loss of tolerance to nuclear antigens, an increase in the proportion of activated T cells and B cells, and mild splenomegaly. These features seem to be linked to a specific haplotype of the signaling lymphocytic activation molecule (SLAM)/CD2 family of genes, indicating that these genes might mediate the autoimmune phenotype associated with the *Sle1b* locus (6,7). However, in the same chromosome 1 region, there are several other candidate genes, such as *Apcs*, *Fcgr2*, *Cd55*, *Pdcd1*, and *Ro* antigen. All of these genes were inactivated in 129-derived embryonic stem cells, and the knockout models displayed a variable degree of autoimmunity (8–12). However, the interpretation of the autoimmune phenotype (13) observed in these gene-targeted models has recently been questioned (14).

Studies by our group and others (8,15–17) have shown that hybrid strains between 129 and C57BL/6 (B6) mice, commonly used in the generation of gene-targeted mice, are spontaneously predisposed to the development of humoral autoimmunity, with low levels of renal disease. Genome-wide linkage studies conducted on the 129 × B6 hybrid mice identified a strong association between a 129-derived segment on chromosome 1, now named *Sle16*, and the expression of autoantibodies (14,18). The autoimmune effect of this locus has recently been defined further by the study of B6 subcongenic strains carrying 129 fragments of different lengths (14,19). The analysis of these lines revealed that a 129 interval between 87.9 cM and 100 cM (D1Mit15 and D1Mit115) was sufficient to induce the autoimmune phenotype, and the congenic line carrying this fragment, named B6.129chr1b, was selected for the present study. It is noteworthy that the B6.129chr1b congenic mice mirror the combination of genes created when a gene in this region is targeted on 129 embryonic stem cells and then backcrossed onto the B6 genetic background.

Immunoglobulin-transgenic models have been instrumental in understanding B cell regulation, revealing several key mechanisms including receptor editing, deletion, anergy, and ignorance (20–24). Chen et al (25) generated an anti-DNA–knockin model in which the rearranged variable heavy chain (V_H_) gene of an anti-DNA antibody (V_H_3H9) was inserted at the *Igh* locus. This allowed the transgenic locus to undergo normal editing, isotype switching, and somatic mutation. A variety of light chains can combine with the V_H_3H9 to yield anti-DNA antibodies (26), but only a few light chains are able to “silence” V_H_3H9 so that it no longer binds to DNA. By virtue of this characteristic, the mice expressing only the V_H_3H9 chain (V_H_3H9R mice) can generate anti-DNA specificities. When the V_H_3H9R mice were crossed with the knockin transgenic light chain Vκ8 mice (27), the antibody generated in the double-transgenic mice (V_H_3H9R/Vκ8R mice) (28) bound only single-stranded DNA (ssDNA) and not double-stranded DNA (dsDNA) (27). Previous studies with the V_H_3H9R mice showed that autoreactive transgenic B cells accumulated in the splenic marginal zone and were regulated by anergy in the presence of a nonautoimmune background, such as BALB/c (25,29) and B6 (30,31), but were activated in a model of chronic graft-versus-host disease (30). Similarly, the V_H_3H9R/Vκ8R-knockin transgenic B cells were regulated by anergy in nonautoimmune disease–prone BALB/c mice, while in autoimmune disease–prone MRL/Mp.*lpr/lpr* animals, transgenic B cells escaped tolerance induction and underwent class switching and affinity maturation (32).

To determine whether the *Sle16* locus could influence the selection of self-reactive B cells, we bred the B6.129chr1b mice with the V_H_3H9R.B6 mice and the V_H_3H9R/Vκ8R.B6 mice and monitored the regulation and activation of anti-DNA–transgenic B cells over a period of 10 months. A similar analysis was also performed with mice deficient in serum amyloid P component (B6. *Apcs*^−/−^), chosen as an example of an autoimmune mouse strain with a targeted deletion of 1 of the candidate genes located within the *129chr1b* region. The analysis of these mice showed that hemizygosity of the *Sle16* locus was sufficient to impair B cell anergy and to induce class switching and epitope spreading. The immunologic alterations were more pronounced in mice that were homozygous for the *Sle16* locus and were similar in the B6. *Apcs*^−/−^ mice, indicating that the lack of serum amyloid P component had no additional effect on the loss of B cell tolerance.

## MATERIALS AND METHODS

### Mice

C57BL/6 mice were obtained from Harlan Olac (Bichester, UK). B6. *Apcs*^−/−^ and B6.129chr1b mice (acknowledged name B6.129-*Sle16*) were generated as previously described (19,33). V_H_3H9R/Vκ8R.B6 mice (25,27) were kindly provided by Prof. M. Weigert (Gwen Knapp Center for Lupus and Immunology Research, University of Chicago, Chicago, IL). In order to generate the different cohorts of mice for study, V_H_3H9R/Vκ8R.B6 mice were crossed with B6. *Apcs*^−/−^ mice, and the resulting V_H_3H9R/Vκ8R.B6. *Apcs*^+/–^ mice were then crossed with B6.*Apcs*^−/−^ mice to obtain V_H_3H9R/Vκ8R.B6. *Apcs*^−/−^ animals. Likewise, V_H_3H9R/Vκ8R.B6 mice were crossed with B6.129chr1b mice, and the resulting V_H_3H9R/Vκ8R.B6129.chr1b^129/B6^ mice were then crossed with B6.129chr1b mice to obtain V_H_3H9R/Vκ8R.B6129.chr1b^129/129^ animals. Only female animals were studied. Mice were bled at regular intervals starting from the age of 2 months and were killed at 10 months of age.

The mice were genotyped by polymerase chain reaction (PCR) analysis, using primers specific for each allele. PCR primers were as follows: for *Apcs* genotyping, Sap5′/9+ (5′-ATTGATTTCCCAGCACAGGGG-3′), Sap8/− (5′-GAT-CAGTTTCAGATTCTCTGG-3′), and neo (5′-GGGGATC-GGCAATAAAAAGAC-3′); for V_H_3H9R genotyping, MW114 (5′-CTGTCAGGAACTGCAGGTAAGG-3′) and MW162 (5′-CATAACATAGGAATATTTACTCCTCGC-3′) (29); for Vκ8R genotyping, MW133 (5′-GGTACCTGTGGG-GACATTGTG-3′) and MW157 (5′-AGCACCGAACGTGA-GAGG-3′) (34); and for *129chr1b* genotyping, D1Mit15 and D1Mit115 (19).

Animals were maintained under specific pathogen-free conditions. All animal care and procedures were conducted according to institutional guidelines and were approved by the local ethics committee.

### Immunofluorescence staining

Flow cytometry was performed using 4-color staining of cells, and the results were analyzed with a FACSCalibur instrument (Becton Dickinson, Mountain View, CA). The following antibodies were used: anti-B220 (clone RA3-6B2), anti-CD4 (clone GK1.5), anti-CD5 (clone 53-7.3), anti-CD11b (clone M1-70), anti-CD19 (clone 1D3), anti-CD23 (clone B3B4), anti-CD21/CD35 (clone 7G6), anti-CD25 (clone PC61), anti-CD69 (clone H1.2F3), anti-CD90.2 (clone 53-2.1), anti-CD138 (clone 281-2), anti-IgM^a^ (clone DS-1). All antibodies were purchased from BD PharMingen (San Diego, CA), with the exception of the anti-V_H_3H9 idiotype (clone 1.209), which was a kind gift from Prof. M. Weigert (35). Biotinylated antibodies were detected using an allophycocyanin-conjugated streptavidin antibody (BD PharMingen). Staining was performed in the presence of a saturating concentration of 2.4G2 monoclonal antibody (anti-FcγRII/III). Data were analyzed using WinMDI software (version 2.8; Scripps Institute and Research Foundation, La Jolla, CA).

Spleens were snap-frozen, sectioned, and fixed in acetone. Sections were stained with fluorescein isothiocyanate (FITC)–conjugated anti-CD90.2, FITC-conjugated peanut agglutinin (PNA) (both from Vector, Peterborough, UK), and biotin-conjugated anti-V_H_3H9 idiotype antibody 1.209, followed with streptavidin–phycoerythrin (BD PharMingen).

### Serologic analyses

Anti-ssDNA, antihistone, antichromatin, and anti-Sm antibodies were measured by enzyme-linked immunosorbent assay (ELISA) as described previously (36). Briefly, the different antigens (DNA from Sigma-Aldrich, Dorset, UK; histone from Roche Diagnostics, Lewes, UK; chromatin from Lorne Laboratories, Reading, UK; and Sm from ImmunoVision, Springdale, AR) were coated onto microtiter plates. Serum samples were diluted appropriately. Bound antibodies were detected with biotinylated mouse anti-mouse IgM^a^ (clone DS-1) and mouse anti-mouse IgG2a^a^ (clone 8.3), followed by a streptavidin–alkaline phosphatase conjugate (all from BD PharMingen). All autoantibody results are expressed in arbitrary ELISA units, as determined by reference to a standard curve derived from serum pools containing high titers of autoantibodies. For total IgM^a^ and IgG2a^a^ determinations, precipitated serum immunoglobulins were coated directly on the microtiter plates and detected as described above.

### Renal assessment

Proteinuria was assessed using Haema-Combistix (Bayer Diagnostics, Newbury, UK). Kidneys were fixed in Bouin's solution and embedded in paraffin, and sections were stained with periodic acid–Schiff reagent. Glomerular histologic features were scored in a blinded manner, using a scale of 0–4 (where 0 = normal and 4 = severe proliferative glomerulonephritis in >90% of glomeruli), as previously described (19). FITC-conjugated goat antibody against mouse total IgG (Sigma-Aldrich) and against mouse C3 (ICN Pharmaceuticals, Costa Mesa, CA) were used to stain snap-frozen sections. Staining with FITC-conjugated antibodies was quantified as previously described (37) and expressed as arbitrary fluorescence units.

### Statistical analysis

The data are presented as the median and range, except where indicated otherwise. The nonparametric Mann-Whitney U test was applied throughout. *P* values less than 0.05 were considered significant. For the findings of the FACS analysis, we applied the analysis of variance Kruskal-Wallis test, followed by Dunn's multiple comparison test. Statistics were calculated using GraphPad Prism software, version 3.0 (GraphPad Software, San Diego, CA).

## RESULTS

### Defective B cell tolerance

**Figure 1:**
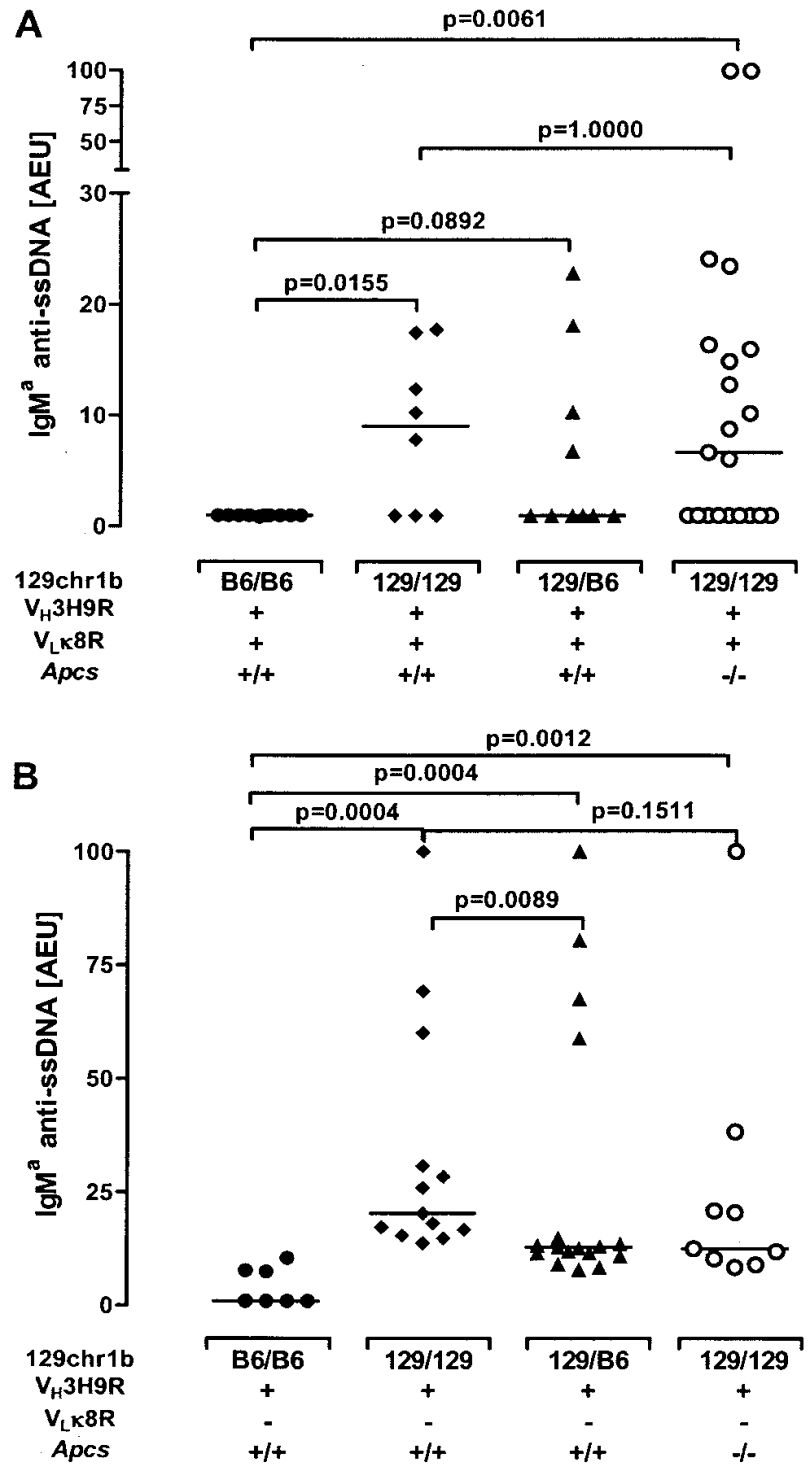
IgM^a^ anti–single-stranded DNA (anti-ssDNA) at 10 months of age in B6 (•), B6.129chr1b^129/129^ (♦), B6.129chr1b^129/B6^ (▴), and B6. *Apcs*^−/−^ (○) mice carrying **A,** the V_H_3H9R/V_L_κ8R transgene or **B,** the V_H_3H9R transgene. Each symbol represents 1 mouse; bars show the median arbitrary enzyme-linked immunosorbent assay units (AEU). Levels of total IgM^a^ were similar in the V_H_3H9R/V_L_κ8R cohorts (median AEU 43 [range 21–112] in V_H_3H9R/V_L_κ8R.B6, 40 [range 16–120] in V_H_3H9R/V_L_κ8R.B6.129chr1b^129/129^, 34 [range 3–65] in V_H_3H9R/V_L_κ8R.B6.129chr1b^129/B6^, and 50 [range 12–160] in V_H_3H9R/V_L_κ8R.B6. *Apcs*^−/−^ mice) and in the V_H_3H9R cohorts (median AEU 67 [range 47–110] in V_H_3H9R.B6, 83 [range 44–102] in V_H_3H9R.B6.129chr1b^129/129^, 60 [range 20–138] in V_H_3H9R.B6.129chr1b^129/B6^, and 74 [range 40–155] in V_H_3H9R.B6. *Apcs*^−/−^ mice). *P* values were determined by Mann-Whitney U test.

B6.129chr1b and B6. *Apcs*^−/−^mice have been reported to develop a lupus-like disease (8,19). To determine the mechanisms underlying the loss of tolerance in these mice, the anti–DNA V_H_3H9R/V_L_κ8R–transgenic alleles were transferred to these mice by breeding. V_H_3H9R/V_L_κ8R.B6.129chr1b^129/B6^ mice carrying only 1 copy of the *129chr1b* allele were also analyzed. The expression of the anti-DNA–transgenic allele was assessed by measuring the transgenic-specific immunoglobulin allotype IgH^a^, since the allotype of endogenous B6 immunoglobulins is IgH^b^ (Figure 1A). As expected, no IgM^a^ anti-ssDNA antibodies were found in the serum of V_H_3H9R/V_L_κ8R.B6 mice. In contrast, the majority of the V_H_3H9R/V_L_κ8R.B6.129chr1b^129/129^ and V_H_3H9R/V_L_κ8R.B6. *Apcs*^−/−^mice produced significant amounts of transgenic anti-ssDNA antibodies. As a result of the gene-targeting technique, B6. *Apcs*^−/−^ mice carry a 129 fragment (168.7–179.4 Mb) of a length similar to that in B6.129chr1b mice (168.7–182.3 Mb). No statistical difference was observed between these 2 groups of mice, indicating that the lack of serum amyloid P component did not contribute to the breakage of B cell tolerance. Of note, 40% of the V_H_3H9R/V_L_κ8R.B6.129chr1b^129/B6^ mice also secreted IgM^a^ anti-ssDNA antibodies, which suggests that hemizygosity in the *129chr1b* region was itself sufficient to break tolerance, although to a lesser extent.

The same analysis was performed in the V_H_3H9R animals. In this model, the 3H9 heavy chain can freely combine with endogenous light chains, giving rise to a limited repertoire of autoantibodies. As previously reported (31), negligible levels of anti-ssDNA antibodies were detected in the sera of V_H_3H9R.B6 mice (Figure 1B). However, the presence of the *129chr1b* locus, even as a single allele, induced significant levels of IgM^a^ anti-ssDNA antibodies. The effect of the *129chr1b* locus was more marked in the homozygous V_H_3H9R.B6.129chr1b^129/129^ mice than in the V_H_3H9R.B6.129chr1b^129/B6^ animals, while the absence of serum amyloid P component had no additional effect. A similar pattern was observed when we measured IgM^a^ antihistone antibodies (data not shown).

### Isotype switching of the transgenic antibodies

**Figure 2:**
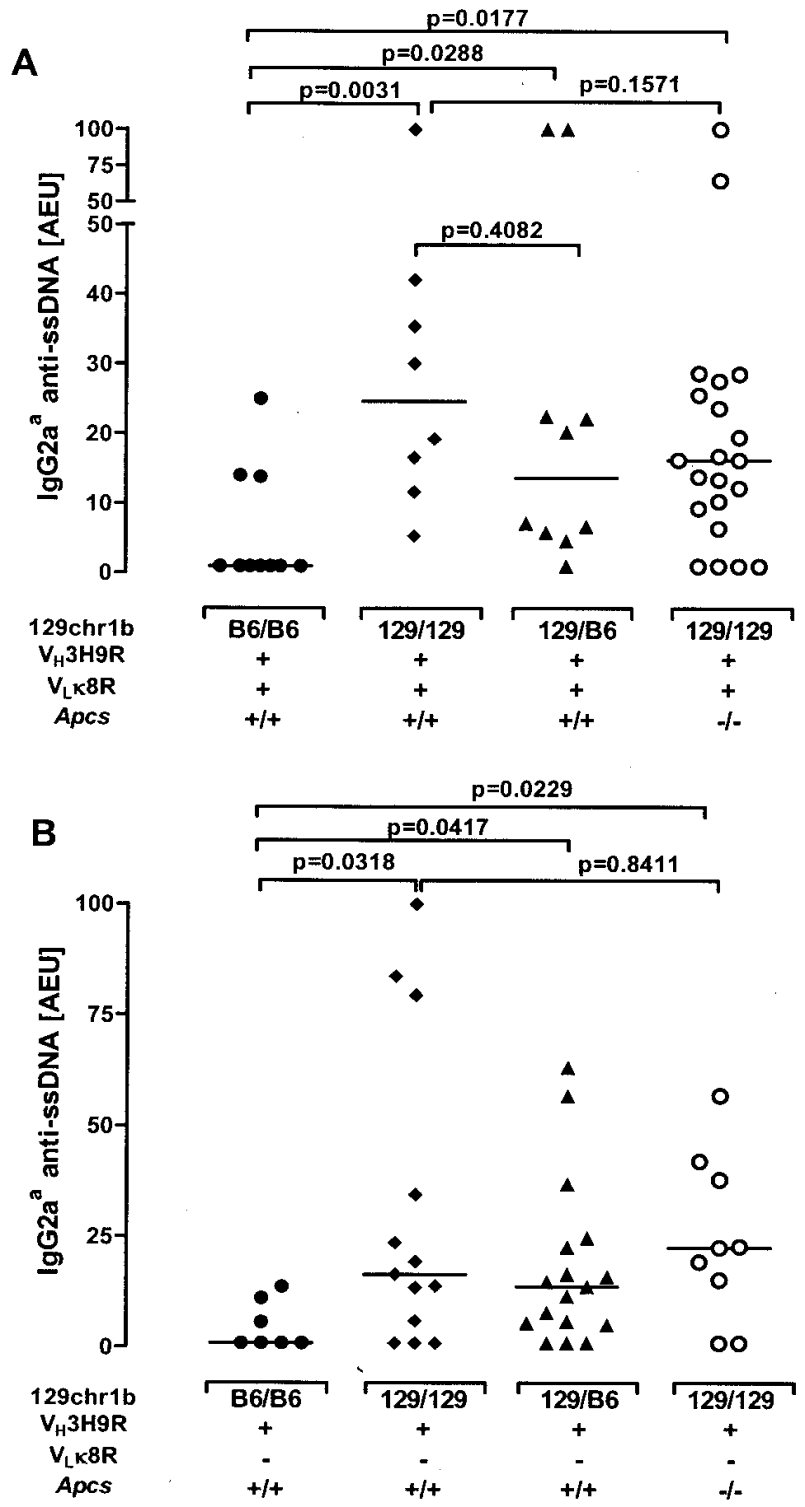
IgG2a^a^ anti–single-stranded DNA (anti-ssDNA) at 10 months of age in B6 (•), B6.129chr1b^129/129^ (♦), B6.129chr1b^129/B6^ (▴), and B6. *Apcs*^−/−^ (○) carrying **A,** the V_H_3H9R/V_L_κ8R transgene or **B,** the V_H_3H9R transgene. Each symbol represents 1 mouse; bars show the median arbitrary enzyme-linked immunosorbent assay units (AEU). Levels of total IgG2a^a^ were similar in the V_H_3H9R/V_L_κ8R cohorts (median AEU 7.8 [range 7.3–9.2] in V_H_3H9R/V_L_κ8R.B6, 7.9 [range 5.8–9.3] in V_H_3H9R/V_L_κ8R.B6.129chr1b^129/129^, 7.5 [range 6.6–8.4] in V_H_3H9R/V_L_κ8R.B6.129chr1b^129/B6^, and 7.5 [range 5.8–9.5] in V_H_3H9R/V_L_κ8R.B6. *Apcs*^−/−^ mice) and in the V_H_3H9R cohorts (mean AEU 7.9 [range 4.7–9.1] in the V_H_3H9R.B6, 8.0 [range 7.3–9.0] in V_H_3H9R.B6.129chr1b^129/129^, 7.5 [range 4.5–9.0] in V_H_3H9R.B6.129chr1b^129/B6^, and 7.9 [range 6.9–8.3] in V_H_3H9R.B6. *Apcs*^−/−^ mice). *P* values were determined by Mann-Whitney U test.

The V_H_3H9R/Vκ8R-knockin model allows the transgenic locus to undergo isotype switching, and we were therefore able to measure IgG2a^a^ anti-ssDNA antibodies. Consistent with the results obtained with the IgM^a^ autoantibodies, markedly increased amounts of IgG2a^a^ anti-ssDNA antibodies were found in the V_H_3H9R/V_L_κ8R.B6.129chr1b^129/129^, V_H_3H9R/V_L_κ8R.B6. *Apcs*^−/−^, and V_H_3H9R/V_L_κ8R.B6.129chr1b^129/B6^ mice as compared with the V_H_3H9R/V_L_κ8R.B6 controls (Figure 2A). Again, there was no statistical difference between the serum amyloid P component–sufficient congenic mice and the serum amyloid P component–deficient animals. Of note, in this assay, the V_H_3H9R/V_L_κ8R.B6.129chr1b^129/B6^ mice had levels of IgG2a^a^ anti-ssDNA antibodies that were similar to those detected in the V_H_3H9R/V_L_κ8R.B6.129chr1b^129/129^ mice.

Similar findings were observed in the V_H_3H9R transgenic mice, with significant levels of IgG2a^a^ anti-ssDNA antibodies in all groups carrying the *129chr1b* locus (V_H_3H9R.B6.129chr1b^129/129^, V_H_3H9R.B6.129chr1b^129/B6^, and V_H_3H9R.B6. *Apcs*^−/−^) (Figure 2B). IgG2a^a^ antihistone antibodies were found in 4 of 13 V_H_3H9R.B6. 129chr1b^129/129^ mice and in 4 of 9 V_H_3H9R.B6. *Apcs*^−/−^ mice, but only in 1 of 17 V_H_3H9R.B6.129chr1b^129/B6^ mice (data not shown). None of the 7 V_H_3H9R.B6 mice had circulating IgG2a^a^ antihistone antibodies.

### Epitope spreading of transgenic B cells

**Figure 3:**
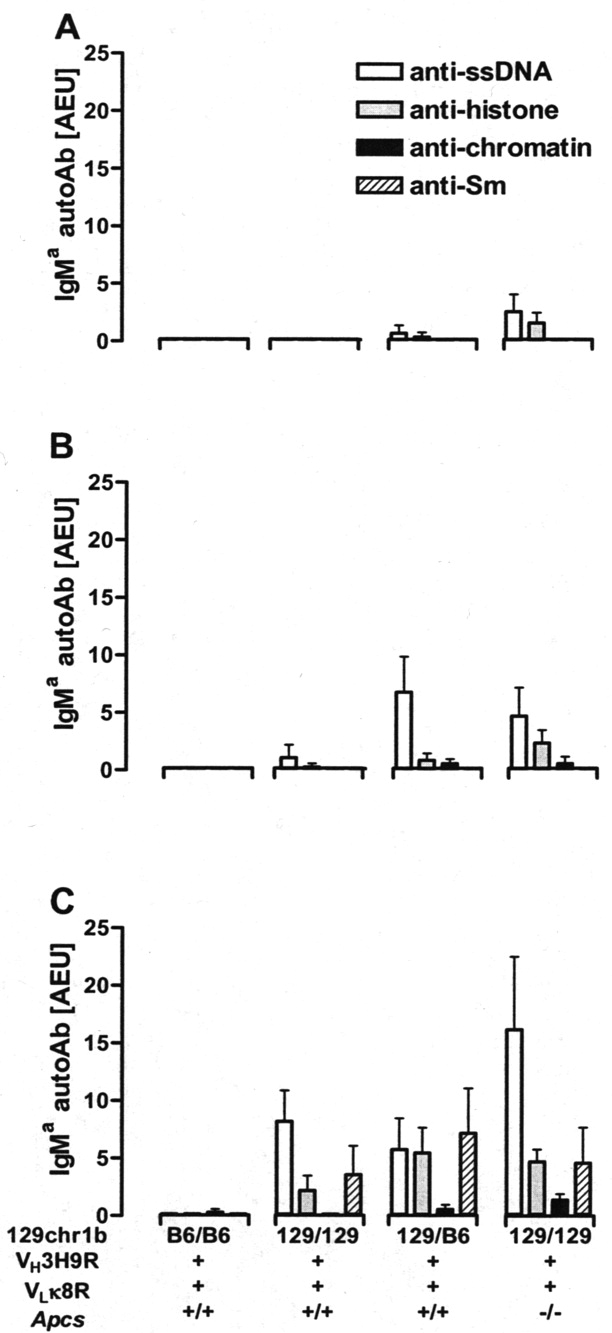
IgM^a^ anti–single-stranded DNA (anti-ssDNA), antihistone, antichromatin, and anti-Sm autoantibodies (autoAb) in V_H_3H9R/V_L_κ8R.B6, V_H_3H9R/V_L_κ8R.B6.129chr1b^129/129^, V_H_3H9R/V_L_κ8R.B6.129chr1b^129/B6^, and V_H_3H9R/V_L_κ8R.B6. *Apcs*^−/−^ mice at ages **A,** 2 months, **B,** 5 months, and **C,** 10 months. Values are the mean and SEM arbitrary enzyme-linked immunosorbent assay units (AEU).

V_H_3H9R/Vκ8R mice can generate only antibodies against ssDNA. However, previous studies of V_H_3H9R/V_L_κ8R.MRL*. lpr/lpr* mice have shown that when tolerance is broken, V_H_3H9R/V_L_κ8R-transgenic B cells can undergo receptor editing in order to change the specificity of the transgenic B cells toward other autoantigens (32). In light of these previous observations, we measured IgM^a^ autoantibodies against different nuclear antigens at different time points in the V_H_3H9R/V_L_κ8R.B6 cohorts (Figure 3). At an early stage of the disease (age 2 months) in mice carrying the *129chr1b* locus, IgM^a^ autoantibodies were mainly directed against ssDNA. However, at later time points (ages 5 months and 10 months), IgM^a^ against other autoantigens (Sm and chromatin) were detectable, indicating an expansion of the autoantibody repertoire.

### Findings of the renal assessment

At 10 months of age, all mice were killed, and their kidneys were harvested and processed for examination by light microscopy (Table 1). Renal sections stained with periodic acid–Schiff were graded in a blinded manner using a scale of 0–4 (19). As expected, neither V_H_3H9R/V_L_κ8R. B6-transgenic nor V_H_3H9R.B6-transgenic mice had significant signs of renal disease. Interestingly, none of the transgenic mice carrying the *129chr1b* fragment developed severe glomerulonephritis, despite having significant titers of autoantibodies in their circulation. Of note, even in the transgenic mice lacking serum amyloid P component, which has been implicated in end-organ injury, there was no histologic evidence of severe renal damage.

**Table 1:** Assessment of renal disease in the different mouse strains*

Mouse strain	GN score	IgG, AFU	C3, AFU
V_H_3H9R/V_L_κ8R.B6	0.0 (0.0–2.0)	18.3 (13.0–29.0)	35.9 (28.2–40.2)
.129chr1b^129/129^	0.0 (0.0–1.0)	24.6 (20.8–27.6)†	32.1 (21.4–40.7)
.129chr1b^129/B6^	0.0 (0.0–2.0)	ND	ND
*.Apcs*^−/−^	0.0 (0.0–1.0)	30.7 (20.0–38.3)†	31.7 (28.4–47.1)
V_H_3H9R.B6	0.0 (0.0–1.0)	19.1 (9.9–26.0)	26.7 (20.4–35.6)
.129chr1b^129/129^	0.0 (0.0–1.0)	36.0 (25.2–58.8)‡	27.8 (19.5–45.4)
.129chr1b^129/B6^	1.0 (0.0–1.0)	ND	ND
*.Apcs*^−/−^	0.0 (0.0–2.0)	27.4 (25.8–35.8)‡	40.0 (27.0–50.5)

* Glomerulonephritis (GN) was graded on a scale of 0–4 as described elsewhere (19). Deposition of IgG and C3 in renal glomeruli was expressed in arbitrary fluorescence units (AFU). In 10-month-old B6 mice (negative controls), the median levels were 19.7 AFU (range 13.6–27.3) for IgG and 32.3 AFU (range 22.8–40.1) for C3, and in nephritic 6-month-old (NZB × NZW)F_1_ mice (positive controls), the median levels were 38.9 AFU (range 31.9–50.9) for IgG and 42.5 AFU (range 41.1–64.2) for C3. Values are the median (range) in 4–7 mice per group. ND = not done.

† *P* < 0.05 versus V_H_3H9R/V_L_κ8R.B6 mice, by Mann-Whitney U test.

‡ *P* < 0.05 versus V_H_3H9R.B6 mice, by Mann-Whitney U test.

We also investigated whether the high levels of circulating autoantibodies were associated with an increased deposition of immune complexes in the kidneys (Table 1). Fluorescence quantification of glomerular IgG deposition revealed a significantly higher amount in V_H_3H9R.B6.129chr1b^129/129^ and V_H_3H9R.B6. *Apcs*^−/−^ mice than in V_H_3H9R.B6 mice (*P* = 0.0006 and *P* = 0.0121, respectively). Similarly, V_H_3H9R/V_L_κ8R.B6.129chr1b^129/129^ and V_H_3H9R/V_L_κ8R.B6. *Apcs*^−/−^ mice had more IgG deposited in their kidneys than did the V_H_3H9R/V_L_κ8R.B6 controls (*P* = 0.0513 and *P* = 0.0140, respectively). Despite the high levels of IgG in their kidneys, the levels of glomerular C3 deposition were similar among the cohorts (for V_H_3H9R.B6.129chr1b^129/129^ and V_H_3H9R.B6. *Apcs*^−/−^ versus V_H_3H9R.B6, *P* = 0.6200 and *P* = 0.0727, respectively; for V_H_3H9R/V_L_κ8R.B6.129chr1b^129/129^ and V_H_3H9R/V_L_κ8R.B6. *Apcs*^−/−^ mice versus V_H_3H9R/V_L_κ8R.B6, *P* = 0.7308 and *P* = 0.8182, respectively).

### Findings of flow cytometric analysis of splenic B cells

In order to determine if the serologic data were accompanied by phenotype changes in T and B lymphocytes, we performed a comprehensive analysis of the splenic subpopulations (Table 2). The proportion of cells expressing the transgenic alleles was first determined by assessing the allotype of their B cell receptor (surface IgM). The great majority of the V_H_3H9R/V_L_κ8R.B6 B cells (>80%) were IgM^a^-positive, indicating that almost all B cells had used the rearranged transgenic alleles. The presence of the *129chr1b* locus did not affect the proportion of transgenic-positive B cells or the level of surface IgM expression (data not shown). The different subpopulations of B cells were then examined. Apart from a significant decrease in marginal zone B cells in V_H_3H9R/V_L_κ8R.B6.129chr1b^129/129^, V_H_3H9R/V_L_κ8R.B6.129chr1b^129/B6^, and V_H_3H9R/V_L_κ8R.B6. *Apcs*^−/−^ mice as compared with the V_H_3H9R/V_L_κ8R.B6 mice (Table 2), no other consistent differences were observed between these 4 experimental cohorts.

**Table 2:** Splenic cell populations in the different mouse strains at 10 months of age*

		Cell type (staining/gate)								
		
				Marginal zone B cells (CD21/35^high^, CD23^low^/CD19+)		Follicular zone B cells (CD21/35+, CD23^high^/CD19+)				
								
Mouse strain	No. of cells (×10^6^)	% transgenic B cells (IgM^a^/B220+)	% T1 B cells, (CD21/35−, CD23−/ CD19+)	%	No. of cells (×10^5^)	%	No. of cells (×10^5^)	% plasmacytes (CD138+/ CD90.2−, CD19−)	% activated T cells (CD69+/ CD4+)	% regulatory T cells (CD25^high^/ CD4+)
V_H_3H9R/V_L_κ8R.B6	319 ± 60	83.8 ± 2.8	14.3 ± 4.2	11.7 ± 1	37 ± 10	63.8 ± 2.9	195 ± 34	4.3 ± 0.4	26.2 ± 3.9	8.0 ± 1.1
.129chr1b^129/129^	153 ± 53	84.7 ± 2.0	13.0 ± 6.4	5.2 ± 0.4†	17 ± 9	77.3 ± 3.1	142 ± 52	4.4 ± 0.6	22.1 ± 6.2	5.2 ± 1.3
.129chr1b^129/B6^	258 ± 122	80.8 ± 4.7	16.7 ± 8.6	5.9 ± 0.8†	8 ± 3	71.4 ± 4.1	102 ± 37	4.0 ± 0.8	16.1 ± 1.5	5.3 ± 1.1
*.Apcs*^−/−^	371 ± 55	89.2 ± 1.1	13.2 ± 7.6	7.0 ± 1.2†	29 ± 5	69.5 ± 4.0	308 ± 43	5.3 ± 0.6	28.7 ± 2.9	7.3 ± 1.2
V_H_3H9R.B6	245 ± 49	87.4 ± 1.9	14.2 ± 4.9	25.3 ± 2.0	62 ± 9	48.2 ± 3.4	116 ± 19	5.1 ± 0.8	25.8 ± 2.6	7.3 ± 0.7
.129chr1b^129/129^	186 ± 39	81.7 ± 5.0	18.0 ± 5.1	22.1 ± 2.0	42 ± 12	49.1 ± 2.2	88 ± 22	5.7 ± 0.6	26.7 ± 3.4	5.4 ± 0.6
.129chr1b^129/B6^	121 ± 29	79.4 ± 4.0	21.3 ± 4.9	23.9 ± 1.6	25 ± 7	44.3 ± 2.8	45 ± 11	4.5 ± 0.8	26.9 ± 3.5	5.8 ± 0.8
*.Apcs*^−/−^	388 ± 51	89.1 ± 1.5	13.2 ± 3.5	21.7 ± 4.2	75 ± 14	46.0 ± 4.8	164 ± 23	6.3 ± 1.2	41.5 ± 1.4	6.8 ± 0.9

Values are the mean ± SEM of at least 7 mice per group. Percentages refer to the percentage of cells stained within the gated population.

*P* < 0.05 versus V_H_3H9R/V_L_κ8R.B6 mice, by Kruskal-Wallis test.

Similarly, in the V_H_3H9R.B6 mice, nearly every B cell was IgM^a^-positive, and the presence of the *129chr1b* allele had no effect on the proportion of B cells expressing the transgenic allele. Consistent with the findings in mice of other genetic backgrounds (38), the V_H_3H9R.B6 mice had more marginal zone B cells compared with the V_H_3H9R/V_L_κ8R.B6 mice (mean ± SEM 25.3 ± 2.0 versus 11.7 ± 1; *P* = 0.0003). However, despite high titers of transgenic autoantibodies, V_H_3H9R.B6.129chr1b^129/129^, V_H_3H9R.B6.129chr1b^129/B6^, and V_H_3H9R.B6. *Apcs*^−/−^ mice displayed a marginal zone of similar size as that of the V_H_3H9R.B6 mice. No substantial differences in the percentages of activated T cells or regulatory T cells were observed in the transgenic animals.

**Figure 4:**
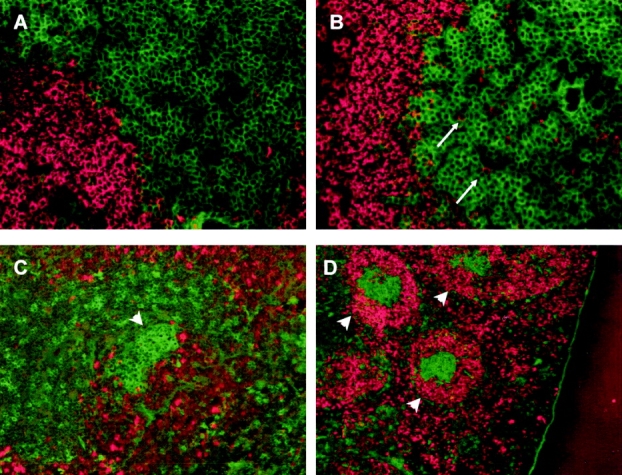
Representative immunostaining of spleen sections from **A** and **C,** V_H_3H9R.B6 mice and **B** and **D,** V_H_3H9R.B6.129chr1b^129/129^ mice. Idiotype-positive B cells (red) localized at the border of the T cell zone (green) in V_H_3H9R.B6 mice (**A**), whereas in V_H_3H9R.B6.129chr1b^129/129^ mice, they were found inside the T cell zone (**B**) **(arrows)**. (Original magnification × 40.) Peanut agglutinin (PNA) staining (green) revealed very few germinal centers **(arrowhead)** in the spleens of V_H_3H9R.B6 mice (**C**), whereas several PNA+ germinal centers **(arrowheads)** surrounded by idiotype-positive B cells (red) were found in the V_H_3H9R.B6.129chr1b^129/129^ mice (**D**). (Original magnification × 10.)

We also explored whether the loss of tolerance was accompanied by alterations of the splenic architecture (39). As previously described in V_H_3H9R.B6 and V_H_3H9R/V_L_κ8R.B6 mice, idiotype-positive B cells remained at the border of the T cell zone (Figure 4A) (30). However, in the V_H_3H9R.B6.129chr1b^129/129^, V_H_3H9R/V_L_κ8R.B6.129chr1b^129/129^, V_H_3H9R.B6. *Apcs*^−/−^, and V_H_3H9R/V_L_κ8R.B6. *Apcs*^−/−^ mice, some idiotype-positive B cells were found inside the T cell zone (Figure 4B). In addition, very few germinal centers were present in spleens from the V_H_3H9R.B6 mice (median of 2 germinal centers per section [range 1–2]) and the V_H_3H9R/V_L_κ8R.B6 mice (median of 1 germinal center per section [range 0–5]), and they did not have a ring of idiotype-positive B cells (Figure 4C). Instead, in the spleens of V_H_3H9R.B6.129chr1b^129/129^ (median of 3 germinal centers per section [range 0–5]), V_H_3H9R/V_L_κ8R.B6.129chr1b^129/129^ (median of 7 germinal centers per section [range 3–11]), V_H_3H9R.B6. *Apcs*^−/−^ (median of 9 germinal centers per section [range 2–12]), and V_H_3H9R/V_L_κ8R.B6. *Apcs*^−/−^ (median of 8 germinal centers per section [range 4–20]) mice, germinal centers were easily detectable (*P* < 0.05) and idiotype-positive B cells surrounded the PNA+ core (Figure 4D).

## DISCUSSION

Multiple genetic loci are known to contribute to the development of SLE. Recently, it was shown that epistatic interactions between 129 and B6 genes, even though neither strain is lupus-prone, can lead to the development of a spontaneous lupus-like disease (8,15–17). Genetic linkage and congenic strain analyses have confirmed that a 129-derived region on chromosome 1 (*Sle16*) is strongly linked to the production of high titers of autoantibodies in the context of a B6 background. In order to elucidate the pathways that contribute to this strain-specific epistasis, we turned to well-established transgenic models of B cells expressing anti-DNA antibodies, the V_H_3H9R/V_L_κ8R.B6- and V_H_3H9R.B6-knockin models.

Several checkpoints present during B cell ontogeny avoid the production of self-reactive autoantibodies. Centrally, most autoreactive specificities are removed by deletion and editing before entering the mature B cell compartment; however, some others are regulated by anergy and exit to the periphery, where they can be rescued (40). Serologic analysis of the V_H_3H9R/V_L_κ8R.B6- and V_H_3H9R.B6-transgenic mice showed that the presence of a single *129chr1b* locus impaired B cell anergy. The anergic B cells may have been rescued by a strong cross-link of the B cell receptor. In this context, one could speculate that the defective uptake of apoptotic cells described in 129chr1b mice (19) might provide a large source of aggregated autoantigens and give a strong signal. In addition, preliminary data suggest that B6.129chr1b B cells are more susceptible to B cell receptor cross-linking.

Another time when autoreactive B cells may arise is during germinal center reactions, when somatic hypermutation occurs. At this checkpoint, B cell survival and priming depend heavily on T cell help, indicating that a considerable role in maintaining B cell tolerance in the periphery is deferred to the T cells (41). Contrary to traditional immunoglobulin-transgenic models, in the V_H_3H9R/V_L_κ8R.B6- and V_H_3H9R.B6-knockin mice, the rearranged V genes are located upstream of the constant region, allowing the transgenic B cells to undergo isotype switching, editing, and somatic mutation, processes that give us insight into the peripheral tolerance in these mice. In both models, the presence of the *129chr1b* locus induced the transgenic B cells to produce IgG2a^a^ anti-ssDNA, indicating that these cells had entered the germinal center, where they received T cell help in order to switch. Consistent with this, immunostaining of spleen sections showed several germinal centers with a ring of idiotype-positive B cells and isolated idiotype-positive B cells in the T cell zone only in mice carrying the *129chr1b* locus. However, in these lupus-prone mice, we did not observe clusters of idiotype-positive B cells to suggest extrafollicular B cell maturation (39).

One essential feature of germinal center reaction is affinity maturation, in which antibodies undergo somatic mutation in order to generate antibodies with higher specificity or antibodies against different antigenic specificities. Interestingly, transgenic IgM^a^ antihistone, antichromatin, and anti-Sm were detected at 10 months of age in the V_H_3H9R/V_L_κ8R.B6 mice carrying the *129chr1b* locus, indicating that the transgenic B cells had undergone somatic diversification either by V-chain replacement or by somatic mutation. This epitope spreading suggests an antigen-driven maturation of the transgenic B cells, and again, inefficiently cleared effete cells might have been the source of these autoantigens (19).

B6.*Sle1b* mice, another well-characterized congenic strain carrying an NZM2410 fragment of chromosome 1, has been shown to break anergy in the sHEL–Ig^HEL^ model (42). The *129chr1b* locus (between 168.7 Mbp and 182.3 Mbp) spans the same chromosome 1 interval encompassed by the *Sle1b* locus (between 171.8 Mbp and 173.1 Mbp) (7). Recent genomic characterization of the *Sle1b* locus has identified a cluster of SLAM/CD2 family genes as the strongest candidate genes for mediating the *Sle1b* autoimmune phenotype (6). Of note, the autoimmune-associated haplotype of the B6.*Sle1b* mice, called SLAM/CD2 haplotype 2, is also present in 129/SvJ mice (6), indicating that these 2 models may share some of the pathways that lead to loss of peripheral tolerance. Reported data indicate that 1 of the SLAM genes, *Ly108*, is associated with a defect in immature B cells, leading possibly to an increase in T1 B cells in the B6.*Sle1b* mouse spleens (42). Similarly, we found a slight increase in the proportion of T1 B cells in the nontransgenic 129chr1b congenic mice (data not shown). However, in the anti-ssDNA–transgenic mice, no consistent expansion of T1 B cells was observed at the time point studied (10 months of age).

B cells that produce potentially pathogenic autoantibodies are thought to home to the marginal zone (38,43), and sequestration to this site is believed to prevent them from entering into the germinal center and developing the properties of pathogenic B cells. Consistent with this idea, in V_H_3H9R/V_L_κ8R.B6 mice carrying the *129chr1b* locus, we detected IgG2a^a^ autoantibodies in the circulation and found that the proportion of marginal zone B cells was markedly reduced compared with that in V_H_3H9R/V_L_κ8R.B6 mice, suggesting that the *129chr1b* locus favored the entry of the autoreactive transgenic B cells into the germinal center. In the single heavy chain–transgenic mice (V_H_3H9R), the marginal zone compartment was enlarged as compared with that in the V_H_3H9R/V_L_κ8R.B6 and B6 mice (present study and ref.^38^). Interestingly, this expansion was comparable in all animals, including those that had broken tolerance (V_H_3H9R.B6.129chr1b^129/129^, V_H_3H9R.B6.129chr1b^129/B6^, and V_H_3H9R.B6. *Apcs*^−/−^ mice), indicating that this may reflect not only trapping of autoreactive B cells, but also some developmental blocking. In this context, it is noteworthy that other single heavy chain–transgenic models have displayed a similar phenotype (44,45).

The entry and survival of B cells through a germinal center reaction depend on T cells. Surprisingly, we failed to detect a significant increase in activated T cells in our transgenic mice. However, we analyzed only a limited number of T cell markers, and this may account for our findings. Recent studies have increasingly emphasized the importance of CD4+,CD25+ regulatory T cells in autoimmune disease. In particular, a subset of regulatory T cells has been identified that localizes to the germinal center and is able to inhibit the production and survival of immunoglobulin as well as activation-induced cytidine deaminase expression by germinal center B cells (46). Moreover, regulatory T cell activity has been shown to be important for preventing DNA-specific autoantibody production that is induced by T helper cells (40). We previously reported a significant decrease in the number of CD4+,CD25+ regulatory T cells in 12-month-old B6.129chr1b mice (19). In the anti-DNA–transgenic 129chr1b mice, we observed a tendency toward fewer regulatory T cells, but this did not reach statistical significance. On the other hand, we have also shown that in B6.129chr1b mice, the CD4+, CD25– T cells were resistant to suppression by regulatory T cells, which suggests a potential new mechanism for the loss of peripheral tolerance in this lupus strain (47).

Taken together, our data suggest that genes within the *129chr1b* locus break B cell tolerance, induce T cells to provide help to B cells undergoing a germinal center reaction, and increase the availability of autoantigens. Interestingly, 1 copy of the *129chr1b* locus was sufficient to induce all these effects, although to a lesser degree than that produced by 2 *129chr1b* alleles. One of the candidate genes within the *Sle16* locus is *Apcs*, which is supported by the observation that B6. *Apcs*^−/−^ mice developed a lupus-like illness with autoantibody production and nephritis. However, these mice carry a 129 fragment that is equivalent to the one present in 129chr1b mice. In our study, anti-DNA–transgenic *Apcs*^−/−^ B cells had impaired induction of anergy similar to that in the transgenic 129chr1b B cells, which further supports the view that this gene is neither the primary initiator of loss of tolerance nor a modifier of humoral autoimmunity.

B6. *Apcs*^−/−^ mice, in contrast to B6.129chr1b mice, have been shown to develop renal inflammation, suggesting that this gene may play a protective role in lupus nephritis (8,19). However, neither V_H_3H9R/V_L_κ8R.B6. *Apcs*^−/−^ nor V_H_3H9R.B6. *Apcs*^−/−^ mice developed kidney disease, despite having high levels of autoantibodies. This may reflect the fact that transgenic autoantibodies are of low affinity and, despite being present in the kidneys, are unable to activate complement and induce tissue damage.

Another candidate gene located within the *129chr1b* locus is *Fcgr2b*. Several studies have linked this gene to SLE (48,49), and Fcgr2b-deficient mice have been shown to develop a lupus-like illness (9). These mice crossed with the V_H_3H9R.B6 mice failed to break B cell tolerance; however, when they were bred with another anti-DNA–transgenic model (V_H_3H9-56R.B6), they displayed a marked increase in levels of IgG anti-DNA (31). Taken together, these observations would suggest that that the *Fcgr2b* gene may act as a weak modifier of disease (31).

In conclusion, genes located within the *129chr1b* locus on a B6 genetic background were able to rescue anergic B cells and to induce class switching and receptor revision, whereas the lack of serum amyloid P component had no additional effect. The immunologic alterations were present even in hemizygotes, indicating that the *Sle16* locus plays a key role in autoimmunity. This study corroborates a note of caution in the interpretation of autoimmune features that are present in mice with targeted genes located in the telomere region of chromosome 1. Further studies will be required to identify the 129- and B6-derived genes whose interaction leads to the development of SLE.

## AUTHOR CONTRIBUTIONS

Dr. Fossati-Jimack had full access to all of the data in the study and takes responsibility for the integrity of the data and the accuracy of the data analysis.

**Study design.** Fossati-Jimack, Walport, Botto.

**Acquisition of data.** Fossati-Jimack, Cortes-Hernandez, Norsworthy, Cook.

**Analysis and interpretation of data.** Fossati-Jimack, Cook, Botto.

**Manuscript preparation.** Fossati-Jimack, Botto.

**Statistical analysis.** Fossati-Jimack.
